# Recognition and Optimization of Ingredients Treating Nitroglycerin-Induced Migraine Rats from Wuzhuyu Decoction

**DOI:** 10.1155/2019/6156754

**Published:** 2019-02-24

**Authors:** Yongsong Xu, Sha Wu, Yanchuan Wu, Muxin Gong, Zhimin Wang

**Affiliations:** ^1^Capital Medical University School of Traditional Chinese Medicine, Beijing Key of TCM Collateral Disease Theory Research, Beijing 100069, China; ^2^Central Laboratory, Xuan Wu Hospital, Capital Medical University, Beijing Geriatric Medical Research Center, Beijing 100053, China; ^3^Institute of Chinese Materia Medica, China Academy of Chinese Medical Sciences, Beijing 100700, China

## Abstract

Wuzhuyu decoction (WZYD) has been clinically used to treat migraine effectively since Eastern Han Dynasty of ancient China. However, its antimigrainic ingredients remain unclear. In present study, the antimigrainic ingredients of WZYD were explored and optimized in nitroglycerin-induced migraine rats through correlation analysis of decoction spectra-pharmacological effects and absorption spectra-pharmacological using entropy-weighted partial least squares regression method. The decoction spectra and absorption spectra were obtained through the determination of nine main ingredients in ten kinds of WZYDs and WZYDs' single-pass intestinal perfusion samples using high performance liquid chromatography-diode array detector. The pharmacodynamics indexes related to migraine model rats were detected using high performance liquid chromatography method and kits after oral administration of WZYDs. Then, the key ingredients influencing indexes were achieved through the correlation analysis. And the optimization of key ingredients was acquired through uniform design experiment. The pharmacodynamic verification test was used to clarify the advantages of the optimized sample. The results showed that the final optimized sample, in which the concentrations of rutaecarpine, evodiamine, ginsendside Rb_1_, 6-gingerol, ginsendside Rg_1_, rutaevine, and limonin were 0.081, 0.565, 1.455, 0.159, 0.871, 0.178, and 0.009 mg·mL^−1^, respectively, provided the best comprehensive effect than another optimized sample and the best uniform design sample. Therefore, a new reliable method for rapidly recognizing and optimizing the effective constituents of WZYD treating migraine was established.

## 1. Introduction

Migraine is a multifactorial primary headache with a prevalence up to 5% worldwide [1]. It was ranked as the seventh-highest cause of disability and third most prevalent disorder [2]. Multiple factors, such as monoamine neurotransmitters and nitric oxide (NO), are involved in migraine [3]. These closely migraine-related indices significantly influence vasoconstriction and vasodilatation [4]. Nowadays, nonsteroidal anti-inflammatory drugs and triptans are prevalent used for migraine clinically [5]. However, some patients are inadmissible to use for several side effects. Gastroduodenal injuries, such as diarrhea, emesis, and even intestinal perforations, are widely recognized after using nonsteroidal anti-inflammatory drugs [6]. And nausea, dizziness and coronary vasoconstriction are also reported after using triptans [7].

Wuzhuyu decoction (WZYD), a classical traditional Chinese medicine (TCM) compound, was initially described in “Shang Han Lun” (Treatise on Exogenous Febrile Disease, written in 219 AD by Zhongjing Zhang). It is clinically used to treat chronic headache and shows significant advantages for migraine with high efficacy [8]. According to previous studies, nine components are detected as the main ingredients of WZYD [9], including evodiamine (Ev), rutaecarpine (Ru), limonin (Li), rutaevin (Rv), limocitrin-glucoside (Lcs), ginsenoside Rb_1_ (Rb_1_), ginsenoside Re (Re), ginsenoside Rg_1_ (Rg_1_), and 6-gingerol (6-Gi). However, several conflicting regulations of these ingredients on the migraine-related factors have been reported. For instance, it is reported that R_g1_ (5 to 500 *μ*g·mL^−1^) dose-dependently reduced 5-hydroxytryptamine (5-HT) release of human platelets [10] while it (6 mg·kg^−1^) upregulated 5-HT level in dementia model mice induced by scopolamine [11]. Similar regulations of 6-Gi on NO are also reported [12, 13]. The different regulations on factors will be more intricate in compounds for interaction between components. For the purpose of getting a better treatment, it is extremely significant to identify the effective ingredients and find out their best ratios in consideration of synergism and antagonism [14].

Therefore, the correlation analysis of spectra-pharmacological effects is utilized to show the relationship between components and efficacy. It is a novel methodology combining the fingerprint studies with pharmacodynamics results using mathematical methods. Presently, it is widely used to screen the effective ingredients in complex Chinese medicines* in vitro* [15] and* in vivo* [16]. An* in vitro *correlation analysis between the contents of nine ingredients in WZYDs and pharmacodynamics results had been performed in reserpine-induced migraine model mice [9, 17]. Four ingredients, including Ev, Rb_1_, Rg_1_, and Ru, were temporarily recognized as the important ingredients due to objective conditions. It is inexhaustible to recognize and optimize all active ingredients in the compound, especially for micro constituents. There are also several limitations on* in vivo* studies, including the high sensitivity and cost of the analytical technique, the omission of possible effective constituents that are not absorbed, the synchronicity of pharmacokinetics and pharmacodynamics due to the complex dynamic internal process [18], and the difficulty in guiding later optimization because the components identified were not original. Therefore, a new strategy* in situ*, which pays more attentions to the limited ingredients in absorption samples, obtained from single-pass intestinal perfusion model, was put forward to help recognized antimigrainic components in WZYD. Meanwhile, in order to further study the therapy of WZYD on migraine with different symptoms, nitroglycerin-induced model rats were used in this research.

In summary, the correlation analysis of intestinal absorption and decoction spectra-pharmacological effects was used to recognize the antimigrainic ingredients from WZYD in nitroglycerin-induced model rats, and uniform design experiment was used to optimize the best concentrations of important ingredients in order to improve the therapy of WZYD and provide a new rapid-recognition and optimization strategy of pharmacological active components in TCM compounds.

## 2. Materials and Methods

### 2.1. Chemicals and Reagents

Chemical reference ginsenoside Rg_1_ (Rg_1_, Lot no. PS100104-03), ginsenoside Re (Re, Lot no. PS160401-154), ginsenoside Rb_1_ (Rb_1_, Lot no. PS161202-04), limonin (Li, Lot no. PS14121501), evodiamine (Ev, Lot no. PS10110101), rutaecarpine (Ru, Lot no. PS11011101), and 6-gingerol (6-Gi, Lot no. PS14032501) were provided by Beijing Yimin Pharmaceutical Co., Ltd. (Beijing, China; Lot no. 20151008). Sumatriptan succinate was provided by Chengdu Push Bio-Technology Co., Ltd. (Chengdu, Sichuan, China). The purity of these reference standards was all more than 95%. Rutaevin (Rv) was provided by Absin Bioscience Co., Ltd. The purity of Rv was more than 98%. Limocitrin-glucoside (Lcs) was produced in Beijing Key Lab of TCM Collateral Disease Theory Research (Beijing, China). The purity of Lcs was more than 95%.The NO assay kit, NOS assay kit, and Coomassie brilliant blue were provided by Nanjing Jiancheng Bioengineering Institute (Nanjing, Jiangsu, China). Nitroglycerin (NTG) injections were provided by Beijing Yimin Pharmaceutical Co., Ltd. (Beijing, China; Lot no. 20151008). Sumatriptan succinate tablets were provided by Tianjin Huajin Pharmaceutical Co., Ltd. (Tianjin, China; Lot no. 2F69151). 5-hydroxytryptamine (5-HT, Lot no. BCBJ6778V), 5-hydroxyindole acetic acid (5-HIAA, Lot no. BCBP2642V), dopamine (DA, Lot no. BCBC4070), norepinephrine (NE, Lot no. 070M1969V), 3,4-dihydroxybenzylamine (3,4-DHBA, Lot no. 16290-26-9), and HPLC-grade methanol and acetonitrile were provided by Thermo Fisher Scientific (Waltham, MA, USA). All other reagents were of analytical grade.

### 2.2. Instruments

The content of nine compounds in WZYDs and absorption samples was determined using an Agilent 1100 series HPLC system (Agilent Technologies, Santa Clara, CA, USA), equipped with a quaternary low-pressure infusion pump, and the content of the monoamine neurotransmitters was detected using an ESA HPLC system (IKA, Staufen, Germany), equipped with a binary low-pressure infusion pump. The NO and NOS activity were determined using UV-1700 ultraviolet visible spectrophotometer (Shimadzu, Japan). The absorption samples were obtained using a BT100-1L peristaltic pump (Baoding Longer Precision Pump Co., Ltd, Hebei, China).

### 2.3. Animals

Male Sprague-Dawley rats (8-9 weeks, 280-320 g) were provided by Vital River Laboratory Animal Technology Co., Ltd. (Beijing, China, License no. SCXK 2016-0011). Animals were housed at controlled room temperature (25±2°C) and relative humidity (50±10%) with standard diet pellets and water* ad libitum* and exposed to 12 h light/dark cycles. At least 4 days of acclimatization were needed before the experiments. All animal procedures were approved by the Beijing Capital Medical University Ethics Committee (Beijing, China, Ethical Approval Number: AEEI-2014-128.)

### 2.4. Preparation of WZYD

Medicinal materials in WZYD, including Euodiae Fructus (dried and nearly ripe fruit of* Euodia ruticarpa* (A. Juss.) Benth.), Ginseng Radix et Rhizoma (fresh root or rhizome of* Panax ginseng* C. A. Mey.), Zingiberis Rhizoma Recens (dried rhizome of* Zingiber officinale* Roscoe.), and Jujubae Fructus (the dried ripe fruit of* Ziziphus jujuba* Mill.), were appraised by associate Professor Luo Rong (School of Traditional Chinese Medicine, Capital Medical University) according to Chinese Pharmacopeia 2015 Edition.

Dry powders of WZYDs, negative control samples, and unoptimized samples (UNOS) were produced according to the previous report [9]. All powders were dissolved with distilled water or K-R solution in different experiments.

### 2.5. Preparation of the Single-Pass Intestinal Perfusion Absorption Samples

To prepare the single-pass intestinal perfusion model, rats were anesthetized with 20% urethane by intraperitoneal injection (1000 mg/kg). The body temperature was kept at 37°C during the operation. The operations were carried out in accordance with the literature [19]. Accurate volume (V_in_ and V_out_), length (a), and width (b) of each segment were measured. Samples (200 *μ*L) were collected and freeze-dried to prepare the test solutions.

The freeze-dried samples were treated by ultrasonic waves for 30 min after mixing for 30 s with 500 *μ*L of methanol. Then, the supernatant was dried under nitrogen after centrifuging at 2,296 × g for 10 min at 4°C. Then, the residues were treated by ultrasonic waves for 5 min after mixing for 30 s with 100 *μ*L of methanol.

The amount of each ingredient in WZYD per area (M_per  area_) was calculated as follows:

(1) $$\begin{array}{c} M_{per\;area}=\frac{v\times \left(C_{in}-C_{out}\times V_{out}/V_{in}\right)\times 15}{a\times b} \end{array}$$

The negative control sample absorption was also determined using the above method.

The composition contents of WZYDs and absorption samples were determined using the previous high performance liquid chromatography-diode array detector method [9].

### 2.6. Preparation of Nitroglycerin-Induced Model Rats

The rats were randomly divided into 13 groups (9 rats in each group). Each group was administered the corresponding drug at a dose of 10 mL/kg body weight, twice a day for 5 consecutive days. The rats in the model group and the normal control group were orally administered with an equal volume of distilled water. Thirty minutes after the last treatment, all rats, except those in the normal control group, were subcutaneously injected with NTG (10 mg/kg) in the frontal region. The rats in the normal control group were injected with an equivalent volume of vehicle.

### 2.7. Sampling

Two hours after model establishment, all rats were anesthetized with 20% urethane by intraperitoneal injection (1000 mg/kg). Blood samples were collected from the abdominal aorta and centrifuged at 2,296 ×*g* for 10 min at 4°C after 2 h of standing at 4°C. Then serum was collected and stored at −20°C for the NO content (NO-p) and NOS activity (NOS-p) assays. Brainstems were separated into two parts along with the center line and stored at −80°C, the left for the NO content (NO-b) and NOS activity (NOS-b) assays and the right for the determination of neurotransmitters.

### 2.8. Content Determination of Monoamine Neurotransmitters

The monoamine neurotransmitters including 5-HT, 5-HIAA, NE, and DA were determined in accordance with method reported [9] using an electrochemical detector.

### 2.9. NO Content and NOS Activity Assay

The NO content and NOS activity in the serum and brain were determined according to the kit instructions.

### 2.10. The Uniform Design Experiments and Verification Test

To find out the best concentrations of the important ingredients (including R_b1_, Rv, Ev, Ru, R_g1_, Li, and 6-Gi) in the spectra-effects correlation, first, the uniform design samples (UDSs) were prepared by blending WZYD with a chemical reference substance according to the criteria. Second, pharmacodynamics indices were obtained using the same methods as described above. Third, several methods such as the entropy-weighted partial least squares regression method, quadratic polynomial stepwise regression method, partial least squares regression method, and stepwise regression method were used for regression modelling. Finally, verification tests for optimized samples (OSs) and unoptimized samples (UNOSs) were performed using the same method as described above.

The UD experimental design is shown in Table 1.

### 2.11. Statistical Analyses

All data were presented as mean ± SEM. SPSS 19.0 statistical software (SPSS, Inc., Chicago, IL, USA) was used to complete statistical analyses. One-way ANOVA followed by the least significant difference test or Tambane's T2 analysis was used to evaluate statistical significance and* P *< 0.05 was considered significant.

### 2.12. Analytical Methods for the Correlation Analysis of Spectra-Pharmacological Effects

Minitab 17.0 R2012b (8.0.0.783) statistical software was adopted to analyse the correlations between the contents of nine ingredients in WZYDs, absorption samples, and pharmacodynamics through the partial least-square regression method, entropy-weighted method. The regression coefficients (RC) were recorded after the explanatory abilities of the predominant components for independent variables and dependent variables were more than 90% by adding factor numbers manually.

Comprehensive evaluation was widely used to assess the impact of a component on the pharmacological effects when multiple indices were detected in a study. Reasonable distribution of the weight is the key to comprehensive evaluation. Therefore, the entropy-weighted (EW) method was utilized via a program written by MATLAB 12.0. It was recognized as a more objective method. Indices were divided into the benefit and cost indices according to the changes after modelling [20], which avoided the subjective differences in different specialists [21]. The values of benefit indices were usually decreased after modelling compared to normal animals; in other words, the higher values of efficiency indices, the better effects, such as 5-HT and 5-HIAA; they were standardized according to (2). Conversely, cost indices, including NE, NO-p, and NO-b, were standardized using (3). The comprehensive effect (CE) of each compound was calculated according to (4). The sign of regression coefficients should be changed in the calculation of comprehensive effects [22].

(2) yij=yij′−mini⁡yij′max⁡yij′−min⁡yij′

(3) yij=yij′−mini⁡yij′max⁡yij′−min⁡yij′

(4) CE=∑1nEWn×RCn

## 3. Results and Discussion

### 3.1. The Determination of 9 Ingredients in WZYD

The result from the determination of 9 ingredients in WZYD is shown in Table 2.

### 3.2. The Absorbed Quantities of 9 Ingredients in WZYD

The result from the determination of the absorbed quantities of 9 ingredients in WZYD is shown in Table 3.

### 3.3. The Pharmacodynamics Results of Screening Experiments

**Figure 1:**
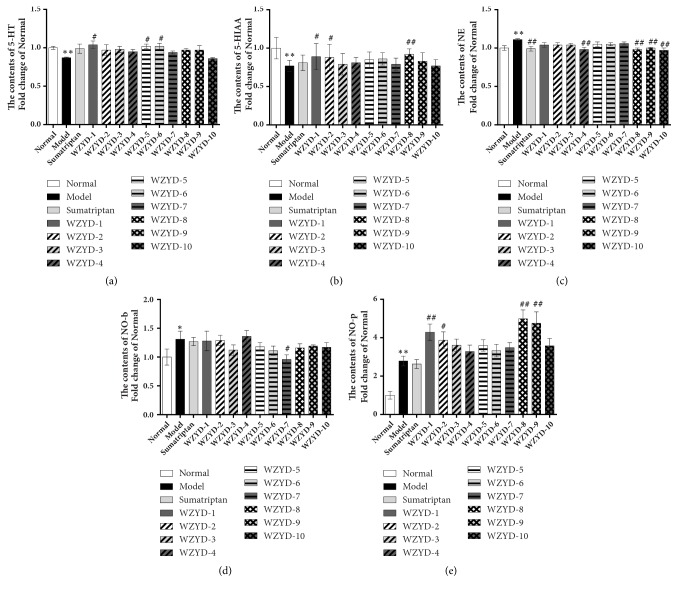
The pharmacodynamic results of screening experiments. (a, b, c) The contents of monoamine neurotransmitters determined by electrochemical detector. (d, e) The contents of NO-b and NO-p determined by NO assay kit. Data are shown as mean ± SEM (Fold change of normal group). *∗P *< 0.05 and *∗∗P *< 0.01 versus Normal group; ^#^*P *< 0.05 and ^##^*P *< 0.01 versus Model group. Data were analysed by one-way ANOVA followed by least significant difference or Tambane's T2 analysis (n = 6 animals per group).

As shown in Figure 1, compared to the normal control, the 5-HT and 5-HIAA contents were significantly decreased and the NE, NO-b, and NO-p contents were significantly increased in the models. 5-HIAA was the metabolite of 5-HT, which reflected the change in 5-HT to some extent [23]. The production and metabolism of 5-HT are dynamic processes of migraine patients, and an increase in the metabolite may also be due to an increase in the prototype. After the administration of WZYD, the 5-HT and 5-HIAA contents were significantly increased, and the NE and NO-b content was reduced while the NO-p content was still increased. The contents of DA, NOS-b, and NOS-p were also determined but there were no noticeable changes.

### 3.4. The Partial Least Squares Regression Analysis Results

The entropy weight of NE, 5-HT, 5-HIAA, NO-p, and NO-b was calculated as 0.29, 0.13, 0.22, 0.20, and 0.16. The regression coefficients between the contents of the nine ingredients in WZYDs and the values of pharmacodynamics indices are presented in Table 4. The positive numbers suggest that the ingredients had positive correlations with single pharmacodynamics index or comprehensive pharmacodynamics indices and vice versa for negative numbers. The greater the absolute value, the stronger the effect. For example, the regression coefficients on 5-HT, which was max in these components, meant that Ev possessed the strongest promotion effect on 5-HT, the same as the comprehensive effect in nine ingredients.

### 3.5. The Pharmacodynamic Results of Uniform Design Experiments

**Figure 2:**
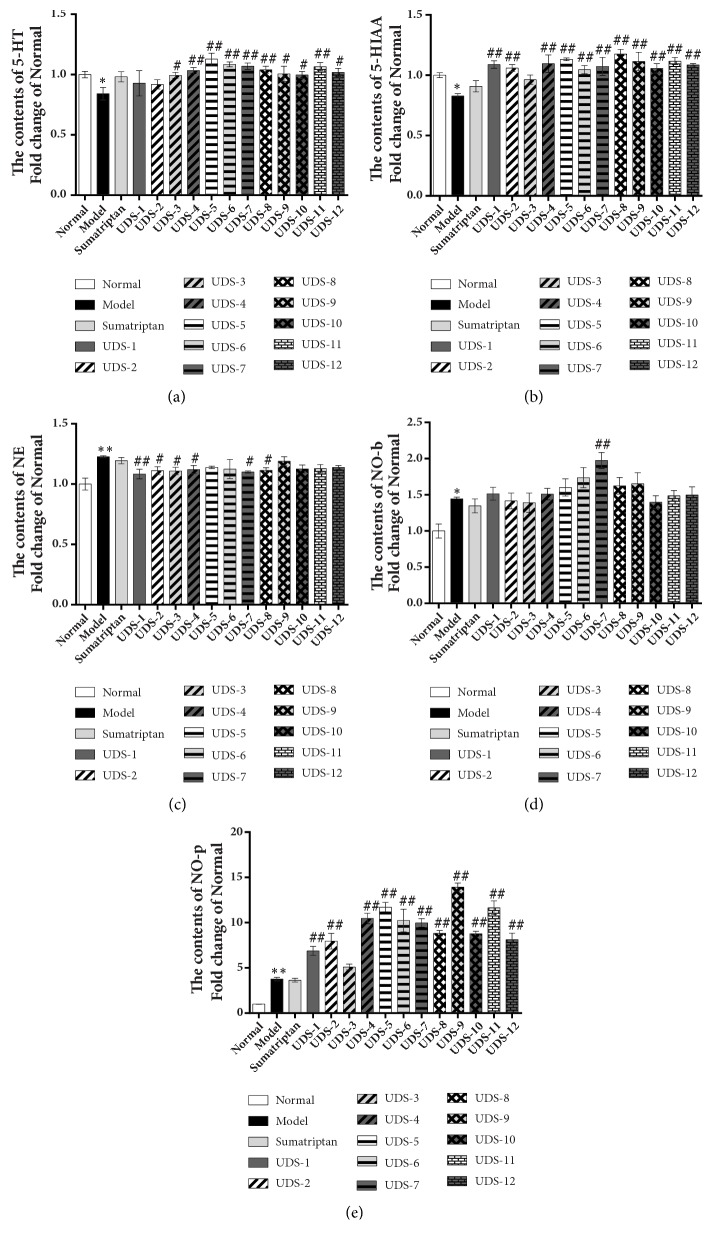
The pharmacodynamic results of uniform design experiments. (a, b, c) The contents of monoamine neurotransmitters determined by electrochemical detector. (d, e) The contents of NO-b and NO-p determined by NO assay kit. Data are shown as mean ± SEM (Fold change of normal group). *∗P *< 0.05 and *∗∗P *< 0.01 versus Normal group; ^#^*P* < 0.05 and ^##^*P *< 0.01 versus Model group. Data were analysed by One-way ANOVA followed by least significant difference or Tambane's T2 analysis (n = 6 animals per group).

As shown in Figure 2, compared to the normal control, the 5-HT and 5-HIAA contents were significantly decreased and the NE, NO-b, and NO-p contents were significantly increased in the models. After the administration of UDS, the 5-HT and 5-HIAA contents were significantly increased, and the NE content was reduced while the NO-p content was still increased, and the NO-b content was increased or decreased.

The dependent variable (Y) was the sum of the products of the index and the entropy weight, and the independent variables (X) were the numerical numbers, quadratic terms and pairwise products of X1 to X7 as shown in Table 4. The partial least squares regression method and quadratic polynomial stepwise regression method were used to find the relationship between the dependent variable and the independent variables. Two regression equations and their max optimum solutions in the experimental concentration range were acquired.

The quadratic polynomial stepwise regression method (input *α*=0.15; output *α*=0.15) is as follows:

(5) Y=355.257+9.8931∗X1−41.421∗X4+1.15362∗X6−2.465∗X1∗X4+61.8517∗X2∗X2−997.599∗X2∗X7+5.42235∗X3∗X6+235.457∗X4∗X5−0.92666∗X6∗X6+4076.46∗X7∗X7

The max optimum solution (OS-1) was X1(Rb_1_) =1.4550, X2(Rv)=0.0090, X3(Ev)=0.5650, X4(Ru)=0.0810, X5(Rg_1_)=0.8710, X6(Li)=1.9640, X7(6-Gi)=0.1590 (mg·mL^−1^).

The partial least squares regression method (p-value was 0.001; the equation was significant) is as follows:

(6) Y=373.505+2.283∗X1−4.547∗X2−4.144∗X3−17.416∗X4+4.862∗X5−0.067∗X6+64.789∗X7−0.599∗X1∗X1−3.484∗X1∗X2−2.173∗X1∗X3+45.916∗X1∗X4+0.385∗X1∗X5+0.479∗X1∗X6+7.975∗X1∗X7−1.350∗X2∗X2−3.265∗X2∗X3−45.672∗X2∗X4−0.887∗X2∗X5+0.518∗X2∗X6+16.394∗X2∗X7+1.633∗X3∗X3+45.661∗X3∗X4+1.415∗X3∗X5−3.231∗X3∗X6+23.210∗X3∗X7−77.557∗X4∗X4+94.578∗X4∗X5−7.037∗X4∗X6+224.531∗X4∗X7+0.232∗X5∗X5−1.395∗X5∗X6+44.305∗X5∗X7+0.095∗X6∗X6+7.391∗X6∗X7+212.584∗X7∗X7

The max optimum solution (OS-2) was X1(Rb_1_)=1.4550, X2(Rv)=0.0090, X3(Ev)=0.5650, X4(Ru)=0.0810, X5(Rg_1_)=0.8710, X6 (Li)=0.1780, X7(6-Gi)=0.1590 (mg·mL^−1^).

Then, the UNOS, OS-1, OS-2, and UDS-10 were prepared and used in the verification test using the same methods above.

### 3.6. The Pharmacodynamic Results of Verification Test

**Figure 3:**
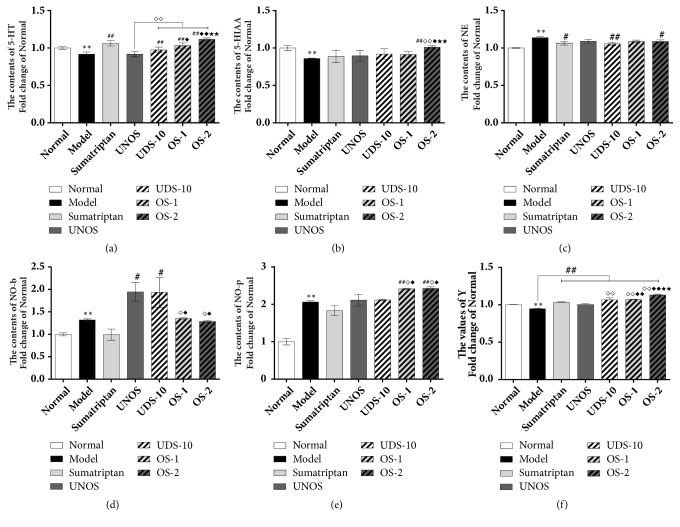
The pharmacodynamic results of uniform verification test. (a, b, c) The contents of monoamine neurotransmitters determined by electrochemical detector. (d, e) The contents of NO-b, NO-p determined by NO assay kit. (f) The Y calculated through the entropy-weighted partial least squares method. Data are shown as mean ± SEM (fold change of normal group). *∗P *< 0.05 and *∗∗P *< 0.01 versus Normal group; ^#^*P *< 0.05 and ^##^*P *< 0.01 versus Model group; ^*◇*^*P *< 0.05 and ^*◇◇*^*P *< 0.01 versus UNOS group; ^*◆*^*P *< 0.05 and ^*◆◆*^*P *< 0.01 versus UDS-10 group; ^★^*P *< 0.05 and ^★★^*P *< 0.01 versus OS-1 group. Data were analysed by One-way ANOVA followed by least significant difference or Tambane's T2 analysis (n = 6 animals per group).

As shown in Figure 3, WZYD significantly ameliorated the decrease in Y induced by the NTG. The Y of OS-2 was more significantly increased than the other samples, which indicated that the comprehensive efficacy of OS-2 was the best. The positive ingredients such as Ru, Ev, R_b1_, and 6-Gi had max concentrations, and the negative ingredients such as Rv and Li had min concentrations, while the negative ingredient R_g1_ also had a max concentration that indicated the complexity of the interactions between compounds.

## 4. Discussion

### 4.1. The Uniform Design Experiment

Uniform design (UD) is a widely used statistical test based on orthogonal design, combined with number theory and multivariate statistics, which is suitable for multiple factors and levels [24]. UD not only reduced the number of investigative factors but also avoided the inclusion of weakly influential variables. The test points may lack representation if using the result of screening test directly for the small differences. In our research, the recognition of antimigraine ingredients was performed through the screening experiments, and the optimization was performed through the UD experiment. Therefore, we paid more attention to the PLS regression coefficients of UD experiment in subsequent analysis, because they were thought to be more credible than that of the screening experiment.

In our UD experiment, the correlation analysis of decoction spectra-pharmacological effects showed that Ru, Ev, Lcs, Rb_1_, 6-Gi, and RE worked positively while Rv, Rg_1_, and Li worked negatively to comprehensive efficacy. The correlation analysis of absorption spectra-pharmacological effects showed that Ru, 6-Go, Rb_1_, Re, Li, and Rv worked positively while Ev, Rg_1_, and Lcs worked negatively to comprehensive efficacy. Taking the decoction-spectrum effects and absorption-spectrum effects into consideration, the most influential positive and negative ingredients were recognized as the important components, including Ru, Ev, Rb_1_, 6-Gi, Rg_1_, Rv, and Li. Lcs was not chosen for the lack of chemical reference substance.

### 4.2. The Choice of the Spectrum Effect Correlation Methods

The PLS regression is a multivariate data analytical technique that generalizes and combines features from principal component analysis and multiple regression [25], providing a regression method for multiple dependent or independent variables, especially suitable for the multicollinearity with independent variables or the samples are less than the number of independent variables [26]. The synthetic action of individual factors on the predictive values is fully considered in the regression. Compared to stepwise regression, it avoids multicollinearity problems, oversimplifications, and overlapping of data [27].

In this study, two kinds of components of the optimal ratio (only the content of Li was different) were verified using the method of PLS regression and stepwise regression analysis. The results showed that the comprehensive efficacy of the optimized decoction was better than stepwise regression according to the results of PLS regression.

In brief, it is necessary for operators to take the requirements and limitations of different statistical methods into account when processing different data. The statistical regression method for the difference between different regression models should be carefully chosen and should be selected strictly. And the excessive dependence on mathematical modelling methods should be avoided. In addition, it is necessary to utilize a certain number of animals to eliminate the impact of individual differences, for ensuring the accuracy and reliability of basic data. The quality of the analysis should be evaluated from the validation to the modelling data and the verification of repeated experiments. The statistical results should be consistent with reality.

### 4.3. The Migraine-Related Pharmacodynamics Indices

Multiple factors, such as 5-HT, 5-HIAA, and NO, were involved in the mechanism of migraine. With migraine attacks, an obvious promotion and reduction of 5-HT were observed in the process. During the migraine aura, the promotion of 5-HT in peripheral blood would cause the vasoconstriction of cerebral vessels, then 5-HT was rapidly metabolized into 5-HIAA, and the sudden reduction of 5-HT would bring out a rebound vasodilation of scalp blood vessels [28]. The promotion of vascular permeability and the exudation of bradykinin or histamine were promoted by 5-HT, which led to perivascular swelling, and then aseptic inflammation was produced and pulsatile headache soon became a persistent migraine attack [29]. 5-HIAA was the metabolite of 5-HT. The increase in 5-HIAA reflected the increasing level or metabolism of 5-HT in a degree.

In the experiments, NTG acted as an NO donor [30]. NO was an important molecule in the regulation of cerebral and extra-cerebral cranial blood flow and arterial diameters in migraine [4]. NO content in the plasma of acute migraine patients was reported to be significantly higher than in normal people (of the same age and sex) with significant differences, which fell to normal levels in remission [31]. In addition, the induction and analgesic effects of pain were reported to be observed in different target cells through the NO-cGMP pathway in the periphery [32]. On the one hand, the algogenic substances and inflammatory mediators easily reached the site of action for the promotion of vascular permeability when the synthesis of NO was in a low level. On the other hand, the transmission of nociceptive information was inhibited as a direct effect on nociceptors when the synthesis of NO was further produced. It was indicated that the affection of different NO levels on pain was different. In this study, the contents of NO-p and NO-b were recognized as cost indices due to the fact that they were increased after modelling.

The contents of DA, NOS-b, and NOS-p were also determined but there were no noticeable changes in the study. Therefore, we paid more attention to the effects of important ingredients on 5-HT, 5-HIAA, NE, NO-p, and NO-b after administration of WZYD as follows.

### 4.4. The Effect of the Important Ingredients on the Pharmacodynamics

The concentrations of Ru, Ev, Rb_1_, 6-Gi, and Rg_1_ were max while Rv and Li were min in the UD experiment, and the comprehensive effect of decoction-spectrum effects was as follows: Ru>Ev>Lcs>Rb_1_>6-Gi>Re>0, 0>Li>Rg_1_>Rv. In other words, the strong positive effects of Ru, Ev, and Rb_1_ on the comprehensive effect were observed, while the negative effects of Rg_1_, Li, and Rv were also observed. The positive effects of 6-Gi and Re were weaker.

The concentrations of Ru, Ev, Rb_1_, 6-Gi, and Rg_1_ were optimized as max. In this study, Ru, Rb_1_, 6-Gi, and Rg_1_ showed positive correlations with 5-HT and 5-HIAA. Although Ev showed a negative correlation with 5-HT, it showed a positive correlation with 5-HIAA. The positive correlations with 5-HT and 5-HIAA of Ev, Rb_1_, and 6-Gi were also observed in another migraine mouse model induced by reserpine [9], indicating that increasing 5-HT and promoting its metabolism may be the main role of these ingredients with max concentrations.

It was pointed out that Ru possesses antiplatelet [33], analgesic [34], and anti-inflammatory [35] activities, inferring that Ru should be the effective component for treating migraine. However, effects of Ru on 5-HT and 5-HIAA were not reported yet. The contents of 5-HT and NE in mouse brains increased through inhibition of monoamine oxidase by Ev (30~120 *μ*M, 9.10~36.40 *μ*g·mL^−1^) [36]. The stress-induced decrease in the levels of monoamines such as 5-HT, 5-HIAA, and DA in the mouse frontal cortex and cerebellum was attenuated by Rb_1_ (10 mg·kg^−1^) [37]. The scopolamine-induced decrease in 5-HT level in the hippocampus of dementia mouse model was inhibited by Rg_1_ (12 mg·kg^−1^) [11]. Moreover, Rg_1_ had neuroprotective effects on dopaminergic neurons in the 6-OHDA model in rats with nigrostriatal injury at a dose of 10 mg·kg^−1^ [38]. In addition, Rg_1_ (1, 10, 100 *μ*M, 0.8, 8, 80 *μ*g·mL^−1^) produced dose-dependent substantial inhibitory effects on the recombinant 5HT (3A) receptor, a receptor related to headache, nausea, and vomiting, in Xenopus laevis oocytes [39], inferring that Rg_1_ should act as an antimigraine ingredient. These previous studies were in agreement with our results.

However, it was reported that 6-Gi (50, 100, and 200 mg·kg^−1^) significantly inhibited the increase of 5-HT and DA levels in the area postrema and ileum of minks induced by the cisplatin [40]. It was distinct from our result, which was possibly related to the different dosages or the presence of other components. Moreover, it was reported that goshuyuto may ameliorate migraine by preventing the hyperaggregation of platelets in migraine with aura for the antiplatelet activity of 6-Gi [41].

In summary, the correlations of Ru, Ev, Rb_1_, 6-Gi, Rg_1_ on 5-HT, and 5-HIAA were essentially positive, but the effects on NO-p and NO-b were complicated as follows. Ev, Rb_1_, and 6-Gi showed positive correlations with NO-p and NO-b, which were also observed in another migraine mice model induced by reserpine [9], indicating that the effect of Ev, Rb_1_, and 6-Gi on NO was stable. Rg_1_ and Ru showed positive correlations with NO-p while negative with NO-b.

There were few studies related to influences of above ingredients on NO in the whole animals. Some studies* in vitro* provided references for us. It was reported that the high glucose-induced decreased NO of human umbilical vein endothelial cells was upregulated by Ev at concentrations from 0.1 to 8 *μ*M (0.03~2.40 *μ*g·mL^−1^) [42]. Rb_1_ exerted vasodilation effects through NO and cyclooxygenase pathways [43]. 6-gingerol ameliorates enhanced vascular contraction in diabetic aortae through increasing production of NO at concentrations from 0.3 to 10 *μ*M (0.088~2.94 *μ*g·mL^−1^) [13]. The lipopolysaccharide-induced increased NO in both cerebral cortex and hippocampus of neurodegenerative mice (NO-b) were inhibited by Rg_1_ (5, 10, and 15 mg·kg^−1^) [44]. The NO level in myocardium of rats was increased by Rg_1_ at dosages of 10 and 15 mg·kg^−1^ [45]. Ru had vasodilator action related to NO [46, 47]. Our results were similar to these reports.

Although the positive effects of above ingredients on NO-p and NO-b were disadvantageous to comprehensive effects, the stronger positive effects on 5-HT and 5-HIAA may lead to a positive comprehensive effect. This may be the reason why the concentrations of Ru, Ev, Rb_1_, 6-Gi, and Rg_1_ were optimized as max.

The concentrations of Li and Rv were optimized as min. In this study, Li showed negative correlations with 5-HT and 5-HIAA; Rv showed negative correlations with 5-HT while positive with 5-HIAA. Li and Rv showed negative correlations with NO-p and NO-b. It was reported that Li (175~400 *μ*M, 82.3~188.2 *μ*g·mL^−1^) inhibited NO production in RAW264.7 macrophages [48]. However, other effects of Li and Rv had not been reported yet. Similarly, the negative effects of Li and Rv on NO-p and NO-b were advantageous to comprehensive effects, while the stronger negative effects on 5-HT may lead to a negative comprehensive effect. This may be for this reason that the concentrations of Li and Rv were optimized as min.

Interestingly, Rv (C_26_H_30_O_9_) and Li (C_26_H_30_O_8_) were two limonoids with similar structures. The carbonyl group was connected to C-6 and the oxhydryl group was connected to C-7 for Rv, while the hydrogen atom was connected to C-6 and the carbonyl group was connected to C-7 for Li.

In summary, max concentrations of Ru, Ev, Rb_1_, and 6-Gi and min concentrations of Rv and Li were consistent with the comprehensive effect of decoction-spectrum effects. The promotion of 5-HT by Ev, the promotion of 5-HT and 5-HIAA by Rb_1_, the promotion of 5-HT, and the inhibition of NO-b by Rg_1_ had been reported, which proved our results. Moreover, it was found that Ru, 6-Gi, and Ev possessed a promotion effect on 5-HIAA, and Ru and 6-Gi possessed a promotion effect on 5-HT. These four ingredients may be the main important components treating migraine with the max concentrations. The inhibitions on 5-HT and 5-HIAA of Rv and Li may be the main reason for the min optimized concentrations.

In short, the effect of a component on an indicator may be affected by concentration, other components or pathophysiological conditions of animals. And it has important significance to improve the therapeutic effect of the compound through clearing the optimal concentrations and the concentrations of components in the animal experiments with other coexisting components. Our results were obtained from the whole animals in the presence of multiple components, which were more consistent with the real situation.

### 4.5. Comparison between Decoction-Spectrum Effects and Absorption-Spectrum Effects

Ev showed a positive effect in the decoction-spectrum effects but showed a negative effect in the absorption-spectrum effects (shown in Table 4), which meant that the higher the content, the better the comprehensive effects, but the better the intestinal absorption, the worse the comprehensive effects. Similar results were found in Rv and Li. These results suggested that the efflux protein or special transport processes may be involved in the intestinal absorption, and it may be affected by the important components selected in our experiments.

P-gp is a drug efflux pump combined with the substrates competitively. WZYD is a complex compound, and we should pay attention to complex interactions of components in absorption, especially with the existence of multiple P-gp substrates or inhibitors.

It was reported that the expression rate of P-gp in mouse models of multidrug resistance of S180 tumour cells was significantly decreased after administration of Evodia rutaecarpa extract at a dosage of 121.6 mg·kg^−1^ [49]. Li [50] and Rb_1_ and Rg_1_ [51] were substrates of P-gp, because the pharmacokinetic behaviour was changed significantly when orally administered with verapamil. Another research reported that although Re (5 *μ*M) showed no inhibitory effect on P-gp activity, their metabolites formed by intestinal bacteria were potent P-gp inhibitors. In addition, the concentration of 6-Gi played an important role in the absorption mechanism; it was not the substrate of P-gp at 30 *μ*M (2.94 *μ*g·mL^−1^) [52] and 50 *μ*M (8.83 *μ*g·mL^−1^) [53] while showing inhibition of P-gp activity at 100, 250, and 500 *μ*M (29.43, 73.59, and 147.18 *μ*g·mL^−1^) [54].

In brief, for the same components, different effects on P-gp were observed, which may be related to the concentration or by different methods such as 6-Gi and Rg_1_. And the metabolites rather than prototypes have inhibitory effects on P-gp such as Rg_1_, Rb_1_, and Re. And the numerous metabolites make it more complicated to combine with P-gp for multiple components [24]. Interaction between components on transport proteins will affect the absorption of components, thereby affecting the curative effect. It is critical to take decoction-spectrum effects and absorption-spectrum effects into consideration. And it is necessary to optimize the best concentrations of important ingredients for a better efficiency. Therefore, we should pay more attention to* in vivo* absorption of pathological models under the whole formula, and the single-pass intestinal perfusion could provide vital reference.

### 4.6. Comparison with Previous Study

Another recognition and optimization of antimigrainic ingredients from Wuzhuyu decoction in reserpine-induced model mice had been performed [9, 17]. Four ingredients, including Ev, Rb_1_, Rg_1_, and Ru, were recognized as the important ingredients. The concentration of Ev was optimized as 312.5 *μ*g·mL^−1^, which was max concentration of the experiment. And the concentration of Rb_1_ was optimized as 1057.4 *μ*g·mL^−1^, which was a middle concentration of the experiment.

These four ingredients were also recognized as the important ingredient in this study, while concentrations of Ev and Rb_1_ were optimized as max. Therefore, Ev and Rb_1_ may be the most influential antimigrainic ingredients in WZYD. The verification test results showed that therapeutic effect of optimized samples were better than that of others. It indicates that the correlation analysis of spectra-pharmacological effects is feasible for the discovery and optimization strategy of pharmacological active components in TCM formulations.

The different optimization results may be involved in the mechanism of the model. For nitroglycerin-induced model rats, nitroglycerin is a pro-drug for NO. NO is an important molecule in the regulation of cerebral and extra-cerebral cranial blood flow and arterial diameters. The absorptions of ingredients in model animals may be increased due to the vasodilatation, change of vascular permeability [55] and inhibition on the expression of P-gp [56] caused by NO, especially for the P-gp substrates. For reserpine-induced model mice, a chronic low dose of reserpine could cause monoamine neurotransmitters exhaustion, sympathetic rejection and parasympathetic excitations, resulting in gastrointestinal peristalsis acceleration, digestive secretion increasing, malabsorption, and weight loss [57]. The absorptions of ingredients in model animals may be reduced for the decrease of ileum villus height and area [58]. The different influence on the absorption may cause different recognition and optimization results. This is why we use different migraine animal models in researches.

Besides, only the positive ingredients were optimized in the previous study due to limitations. In this study, the positive and negative ingredients were all selected as the important ingredients, such as Li and Rv. Although, concentrations of Li and Rv were optimized as min, it still possesses some positive affections. For example, Li and Rv could downregulated the content of NO-p and NO-b, while ingredients with max concentrations usually upregulated these indices. It indicates that Li and Rv are not completely useless or harmful for therapy. So, it is necessary to optimize their concentrations.

In summary, traditional Chinese medicine is a complex system of components. Multitargets and multiinteractions of each ingredient are often the basis of its function, which make it hard to recognize the pharmacodynamic material basis, especially in different animal models. Our researches will provide a new rapid-discovery and optimization strategy of pharmacological active components in TCM formulations.

## 5. Conclusions

A new method for rapidly recognizing and optimizing the effective constituents of WZYD treating migraine through correlation analysis of decoction spectra-pharmacological effects as well as intestinal absorption spectra-pharmacological effects was established. The important ingredients were selected through the correlation between the decoction-spectrum, absorption-spectrum, and pharmacodynamics indexes, and their concentrations were optimized through the uniform design, and the results were proven to be reliable.

Further, more attention will be paid to the difference in pharmacokinetics between optimized and unoptimized samples in model animals to explain the better pharmacodynamics.

## Figures and Tables

**Table 1:** The experimental design schemes and the concentrations of ingredients in UDS and OS (mg·mL^−1^).

Decoction	X1	X2	X3	X4	X5	X6	X7
	R_b1_	Rv	Ev	Ru	R_g1_	Li	6-Gi
UDS-1	0.059	0.078	0.279	0.055	0.639	3.620	0.030
UDS-2	0.186	0.216	0.565	0.023	0.329	2.473	0.037
UDS-3	0.313	0.354	0.231	0.074	0.019	1.325	0.060
UDS-4	0.440	0.493	0.517	0.042	0.717	0.178	0.101
UDS-5	0.567	0.631	0.183	0.010	0.407	4.003	0.159
UDS-6	0.694	0.769	0.470	0.061	0.096	2.855	0.147
UDS-7	0.821	0.009	0.136	0.029	0.794	1.708	0.043
UDS-8	0.948	0.147	0.422	0.080	0.484	0.560	0.036
UDS-9	1.074	0.285	0.088	0.048	0.174	4.385	0.120
UDS-10	1.201	0.424	0.374	0.017	0.872	3.238	0.079
UDS-11	1.328	0.562	0.040	0.068	0.562	2.090	0.113
UDS-12	1.455	0.700	0.326	0.036	0.251	0.943	0.103
OS-1	1.455	0.009	0.565	0.081	0.871	1.964	0.159
OS-2	1.455	0.009	0.565	0.081	0.871	0.178	0.159

**Table 2:** The composition and crude drug concentrations of the WZYD.

Group	Drug concentration	Lcs	R_g1_	R_e_	R_b1_	Rv	Li	6-Gi	Ev	Ru
	g·mL^−1^	mg·mL^−1^								
WZYD-1	0.70	0.123	0.389	0.375	0.769	0.260	0.647	0.138	0.196	0.038
WZYD-2	0.70	0.153	0.089	0.079	0.543	0.211	2.048	0.179	0.111	0.026
WZYD-3	0.35	0.146	0.218	0.220	0.314	0.140	0.384	0.088	0.113	0.018
WZYD-4	0.35	0.010	0.019	0.044	0.116	0.009	1.082	0.059	0.061	0.014
WZYD-5	0.70	0.271	0.373	0.533	0.406	0.208	0.514	0.169	0.115	0.054
WZYD-6	0.70	0.313	0.387	0.404	0.447	0.387	1.235	0.172	0.314	0.047
WZYD-7	0.35	0.224	0.344	0.106	0.059	0.066	0.178	0.074	0.077	0.018
WZYD-8	1.40	0.557	0.841	0.990	0.875	0.769	3.401	0.323	0.565	0.080
WZYD-9	1.40	0.271	0.872	0.598	1.455	0.351	4.385	0.267	0.230	0.055
WZYD-10	0.35	0.011	0.033	0.061	0.143	0.011	1.025	0.052	0.040	0.010
UNOS	0.70	0.125	0.329	0.095	0.109	0.107	0.069	0.046	0.035	0.017

**Table 3:** The absorbed quantities of 9 ingredients in WZYD $(\overset{-}{X}\pm S$, n=6, *μ*g·cm^−2^).

Group	Lcs	R_g1_	R_e_	R_b1_	Rv	Li	6-Gi	Ev	Ru
WZYD-1	4.29±2.47	2.94±1.38	1.59±1.04	2.10±2.30	3.80±0.93	2.79±0.80	0.81±0.20	0.47±0.12	0.29±0.06
WZYD-2	7.75±3.36	1.63±0.85	0.58±0.40	2.10±0.45	2.10±0.32	2.67±0.57	0.95±0.09	0.19±0.10	0.05±0.01
WZYD-3	4.82±1.85	3.18±1.29	2.11±0.38	0.90±0.56	2.10±0.59	0.90±0.26	0.45±0.08	0.10±0.04	0.05±0.01
WZYD-4	1.90±0.78	0.56±0.19	0.73±0.24	0.30±0.21	0.05±0.02	0.53±0.20	0.59±0.18	0.08±0.10	0.03±0.01
WZYD-5	10.16±2.95	3.55±1.35	1.59±0.92	1.50±0.81	1.45±0.39	2.39±0.40	0.62±0.12	0.08±0.02	0.07±0.03
WZYD-6	0.39±0.22	3.17±0.81	2.10±0.57	2.34±1.19	4.21±0.94	3.04±0.53	0.54±0.10	0.52±0.15	0.11±0.02
WZYD-7	3.32±1.30	3.17±0.53	1.12±0.15	0.74±0.21	0.66±0.18	0.26±0.08	0.40±0.05	0.06±0.01	0.04±0.01
WZYD-8	5.20±3.88	2.50±1.08	0.38±0.35	2.12±1.34	2.72±1.42	1.12±0.96	0.27±0.15	0.09±0.07	0.10±0.04
WZYD-9	18.21±9.48	16.93±7.12	10.81±4.03	5.77±3.70	4.21±2.99	6.61±4.81	0.89±0.53	0.34±0.27	0.14±0.14
WZYD-10	1.39±0.45	1.82±0.24	0.46±0.06	1.02±0.37	0.49±0.06	0.41±0.25	0.43±0.11	0.09±0.01	0.03±0.00

**Table 4:** The regression coefficients of partial least squares regression between the contents of the nine ingredients in WZYD and the values of pharmacodynamic indices and the comprehensive effect between decoction spectrum, absorption spectrum, and pharmacodynamic effects.

Ingredient	Unnormalized coefficients					Comprehensive effect	
	NE*∗*	5-HT	5-HIAA	NO-p*∗*	NO-b*∗*	Decoction spectra-effects	Absorption spectra-effects
Lcs	68.92	283.04	-16.91	-36.91	-2.87	21.77	-2.33
Rg1	4.24	-162.32	-27.68	12.66	-1.46	-31.25	-5.64
Re	-38.73	8.31	-6.16	26.65	0.47	5.69	5.85
Rb1	21.08	135.46	9.91	11.03	0.34	11.82	6.01
Rv	7.69	-537.48	26.35	35.12	0.34	-75.33	1.82
Li	-10.35	-19.46	-2.88	1.41	0.04	-0.49	5.40
6-Gi	73.89	167.61	65.15	35.72	1.48	7.60	6.73
Ev	-35.77	524.10	20.20	12.37	0.35	82.31	-71.36
Ru	-14.12	345.56	203.52	-501.13	11.40	193.08	36.92

Notes: *∗*the cost indices including NE, NO-p, and NO-b that needed to change the sign.

## Data Availability

The data used to support the findings of this study are available from the corresponding author upon request.
